# The Regulatory Role of *Cg*ALDH6A1 in the Oxidative Stress Response of *Crassostrea gigas* Under High-Temperature Stress

**DOI:** 10.3390/antiox14121423

**Published:** 2025-11-27

**Authors:** Xingyi Feng, Lei Gao, Hairu Xu, Jiayu Ye, Liming Pang, Lingling Wang, Linsheng Song

**Affiliations:** 1 Liaoning Key Laboratory of Marine Animal Immunology and Disease Control, Dalian Ocean University, Dalian 116023, China; fengxingyi427@yeah.net (X.F.); xuhairu27@yeah.net (H.X.); yejiayu1111@yeah.net (J.Y.); pang_0724@proton.me (L.P.); wanglingling@dlou.edu.cn (L.W.); 2 Dalian Key Laboratory of Aquatic Animal Disease Prevention and Control, Dalian Ocean University, Dalian 116023, China; 3 Laboratory for Marine Fisheries Science and Food Production Processes, Qingdao Marine Science and Technology Center, Qingdao 266237, China

**Keywords:** *Cg*ALDH6A1, MDA, high-temperature stress, the Pacific oyster *Crassostrea gigas*

## Abstract

High temperatures induce oxidative stress and the production of a large amount of malondialdehyde (MDA) in the Pacific oyster *Crassostrea gigas*, and they can even lead to mass mortality. Aldehyde dehydrogenase (ALDH) degrades MDA and is attracting increasing attention for its role in enhancing antioxidant defense capacity. This study identified 14 ALDH family members in the oyster genome. Among them, *Cg*ALDH6A1 harbored a conserved ALDH_F6_MMSDH domain (known to catalyze the oxidation of aliphatic and aromatic aldehydes) and was likely involved in the high-temperature stress response through the detoxification of accumulated toxic aldehydes. In the gills, *Cg*ALDH6A1 had significantly higher mRNA expression than other tissues, with a significant increase at 12 h under 28 °C high-temperature stress. During the outdoor aquaculture period, the mRNA transcripts of *Cg*ALDH6A1 in the gills exhibited a significant increase from June to October. After the expression of *Cg*ALDH6A1 was inhibited by RNAi, the MDA content in the gills increased significantly (1.31-fold, *p* < 0.01), while the activities of superoxide dismutase (SOD) (0.93-fold, *p* < 0.05) and catalase (CAT) (0.45-fold, *p* < 0.001) and total antioxidant capacity (T-AOC) (0.54-fold, *p* < 0.01) decreased significantly under high-temperature stress. Meanwhile, the gill tissue was observed to be disorganized with obvious filament swelling. After the oysters were treated with *Cg*ALDH6A1 agonist (Alda-1), the MDA content (0.59-fold, *p* < 0.001) in the gills decreased significantly, while the activities of SOD (1.33-fold, *p* < 0.001), CAT (1.81-fold, *p* < 0.001), and T-AOC (1.79-fold, *p* < 0.01) all increased significantly 48 h after high-temperature stress. However, no obvious morphological changes were observed in the gills. These results demonstrate that *Cg*ALDH6A1 plays a key role in regulating the oxidative stress response by degrading MDA under high-temperature stress and plays a cooperative role with the antioxidant system in alleviating oxidative stress under high-temperature stress.

## 1. Introduction

The aldehyde dehydrogenase (ALDH) enzymes constitute a superfamily of enzymes that catalyze the oxidation of diverse aldehydes into their respective carboxylic acid products. ALDH catalysts facilitate the irreversible oxidation of internally produced aldehydes, thereby mitigating aldehyde-induced cytotoxicity [1,2,3,4]. They are integral to antioxidant defense mechanisms [5,6,7], notably because of oxidizing lipid peroxidation byproducts [8], mitigating aldehyde-mediated oxidative damage. Studies have shown that high-temperature stress significantly increases malondialdehyde (MDA) production [9,10,11] and triggers oxidative damage in marine organisms [12,13,14]. The investigation of ALDH in the oxidative stress response of marine organisms under high-temperature stress may provide fundamental insights into the high-temperature adaptation mechanisms of marine organisms.

The composition and structure of ALDH have undergone significant changes over the course of evolution. Nevertheless, ALDHs generally possess a conserved structural fold and employ a shared catalytic mechanism—one that incorporates a catalytic cysteine residue and a cofactor (e.g., NAD^+^ or NADP^+^). The traditional ALDH fold consists of four structural components: a conserved foundational structural unit, a Rossmann binding domain, a catalytic domain, and an extra oligomerization domain. The majority of ALDHs adopt a unique twisted dimerization pattern driven by the oligomerization domain; these dimers further assemble regularly to form higher-order complexes, typically tetramers or hexamers. ALDH6A1 functions as a mitochondrial tetrameric protein and is further identified as methylmalonate semialdehyde (MMS) dehydrogenase, with the alternative name MMSDH [15]. ALDH6A1 contains a unique conserved ALDH_F6_MMSDH domain. Being the only identified Coenzyme A (CoA)-dependent aldehyde dehydrogenase in humans, it participates in the catabolism of valine and pyrimidines. By metabolizing malonate semialdehyde, ALDH6A1 participates in the process of converting MDA into acetyl-CoA [16]. Stress conditions elevate levels of MDA, a final product of lipid peroxidation [2,3]. MDA is initially oxidized into acetaldehyde by ALDH and then converted into acetate and finally carbon dioxide and water [17]. Therefore, ALDH plays a critical role in alleviating oxidative damage under environmental stress [18,19]. The ALDH family is widely distributed across organisms and classified into 24 different families (ALDH1~ALDH24) [20], 7 of which are plant-specific: ALDH11, 12, 19, 21, 22, 23, and 24 [21]. Up to now, 19 ALDH genes have been characterized in humans (*Homo sapiens*) [15], and they exert significant effects on biosynthesis and antioxidant defense pathways [22]. Among invertebrates, 11 ALDHs have been identified in the fruit fly, *Drosophila melanogaster* [23,24]. To date, research on ALDH in shellfish remains relatively limited, with 16 ALDH genes having been recognized in the razor clam *Sinonovacula constricta* [25].

Excessive accumulation of aldehyde compounds occurs under high-temperature stress [26,27], which can interact with proteins and nucleic acids, damaging their functions and thereby causing cell death [28]. ALDHs respond to high-temperature stress by modulating their mRNA expression levels and alleviating cell damage induced by aldehydes, thereby alleviating oxidative stress. The transcription levels of the genes ALDH3I1 and ALDH7B4 were significantly increased in mouse-ear cress *Arabidopsis thaliana* after high-temperature stress [29]. An increase in total ALDH activity was observed in the Antarctic microalga *Chlorella vulgaris* under high-temperature stress, indicating that ALDH5 and ALDH6 isozymes play important roles in heat tolerance [30]. Studies on the reaction of ALDH to high-temperature stress in invertebrates remain limited. Research has shown that the expression of ALDH is significantly upregulated in the Pacific white shrimp *Litopenaeus vannamei* under high-temperature stress, indicating that ALDH has a significant function under high-temperature stress [31]. For instance, ALDH2 knockdown resulted in the accumulation of 4-hydroxynonenal (4-HNE) and MDA under high-temperature stress, while Alda-1 (a known activator of ALDH2) alleviated the activation of inflammatory pathways that was induced by high-temperature stress in mice (*Mus musculus*) [32]. The information above remains largely unexplored in regard to invertebrates. Furthermore, the mechanism by which ALDH alleviates oxidative stress in oysters through the clearance of MDA remains poorly understood.

As a commercial bivalve, the Pacific oyster *Crassostrea gigas* is a leading commercial bivalve globally, being cultivated in the highest yields in the world [33,34,35]. Recent research has reported frequent summer mortality events in oyster aquaculture [36,37,38], with elevated temperatures identified as the critical contributing factor [39,40]. High-temperature stress leads to the accumulation of MDA in oysters, thereby triggering oxidative damage and even death [10]. Current research on MDA clearance in oysters primarily focuses on traditional antioxidant systems. ALDH has been demonstrated to play a crucial role in the oxidative stress caused by high temperature in animals. This study investigates the gene structure, phylogenetic relationships, and function of *Cg*ALDH in response to high-temperature stress, aiming to (1) analyze the evolutionary and molecular characteristics of ALDH in oysters, (2) determine the expression patterns of ALDH under high-temperature stress, and (3) clarify the regulatory function of ALDH in antioxidant defense mechanisms.

## 2. Materials and Methods

### 2.1. Experimental Animal

Adult oysters, with an average shell length of 120 ± 20 mm, were collected from a local farm (seawater temperature 18.1 °C) in Dalian, China, in October 2024. Prior to the experiment, a narrow notch near the adductor muscle was cut to facilitate subsequent injection, and the oysters were acclimated in aerated seawater at 15 °C at a salinity of 29–31 for one week. During acclimation, they were fed algae powder once daily, and 50% of the seawater was replaced every two days. All high-temperature-stress treatments in the experiment were conducted by directly exposing oysters to the designated temperature conditions. This study was approved by the Institutional Animal Care and Use Committee (IACUC) of Dalian Ocean University (Approval Number: SHOU-23-014), with approval granted on 15 November 2024.

### 2.2. Bioinformatics Analysis and Identification of CgALDHs

The ALDH sequence data from model organisms (Table S1) were employed as queries to retrieve homologous sequences in the oyster genome databases (GenBank Bioproject IDs: PRJNA276446 and PRJNA598006) via tblastn analysis. For domain analysis, two approaches were employed: SMART (http://smart.embl-heidelberg.de/ accessed on 20 March 2025) [41] was used for initial domain prediction, and conserved domains were further identified via NCBI (https://www.ncbi.nlm.nih.gov accessed on 20 March 2025); meanwhile, conserved motifs were recognized using MEME (https://meme-suite.org/meme/tools/meme accessed on 20 March 2025) [42]. MEGA-X software (version number: v12.0.14) was used for phylogenetic tree construction via the Neighbor-Joining (NJ) method with 1000 bootstrap replicates [43]. Final tree visualization was refined using ChiPlot (https://www.chiplot.online accessed on 21 March 2025) [44]. For the identification of the *Cg*ALDH6A1 sequence, Primer 5 software was used to design the cloning primers *Cg*ALDH6A1-F and *Cg*ALDH6A1-R for the ORF frame (Table 1). The Takara LA Taq enzyme system amplified the ORF sequence of *Cg*ALDH6A1 via PCR. After the PCR product was purified via gel electrophoresis, it was inserted into the pMD19-T cloning vector (Takara, Shiga, Japan) and transformed into competent *Escherichia coli* Trans5α cells for sequencing verification.

### 2.3. Transcriptomic Sequencing and mRNA Expression Under Different Conditions

Twenty-seven oysters were randomly selected for high-temperature stress treatment. Nine oysters served as the untreated control group (AC). Gill tissues were collected from nine oysters at 12 h after subjection to 28 °C high-temperature stress (AH group), and gill tissues were collected from the other nine oysters at 12 h under 35 °C high-temperature stress (AHH group). Total RNA was extracted from all samples for transcriptomic analysis. The cDNA libraries were constructed and sequenced using the Illumina NovaSeq 6000 platform (Illumina, San Diego, CA, USA) to generate paired-end reads. The clean reads were obtained by removing the reads with adapters, the reads with >10% unknown bases, and low-quality reads (reads with >50% of the total length with Qphred ≤ 20). Filtered reads were mapped to the genome using Hisat2 v2.0.5. Transcriptome data were used for the analysis of mRNA expression under high-temperature stress via TBtools (version number: v2.310) [45].

Six ALDHs genes were identified to respond to high temperatures based on transcriptomic data. To investigate the tissue-specific expression patterns of ALDHs in oysters under high-temperature stress, hepatopancreas, hemolymph, gills, mantle, labial palp, gonads, and adductor muscle were collected 24 h after the oysters underwent high-temperature stress at 28 °C; here, 28 °C was used for high-temperature exposure because it is the highest temperature recorded in the North Yellow Sea [46]. The tissues from three oysters were pooled together as one sample, and there were three samples for each tissue. Total RNA was isolated from all samples to determine the mRNA expression levels of *Cg*ALDH1β1, *Cg*ALDH1β2, *Cg*ALDH2α, *Cg*ALDH6A1, *Cg*ALDH7A1, and *Cg*ALDH8A1. Tissue-specific expression analysis revealed that three ALDH genes exhibited the highest expression levels in the gills. Given that gill tissue is the primary tissue involved in the response to high-temperature stress, these three genes were selected for time-course expression analysis. To stimulate the long-term high-temperature stress that oysters experience in summer, 72 oysters were exposed to high-temperature stress at 28 °C, with 9 oysters randomly collected at 0 h, 12 h, 24 h, 48 h, 72 h, 7 d, 14 d and 21 d after being subjected to high-temperature stress. Total RNA was isolated from all samples to determine the mRNA expression levels of *Cg*ALDHs.

Given that *Cg*ALDH6A1 responds to high temperatures, an outdoor aquaculture experiment was conducted to analyze its expression pattern. The aquaculture investigation in this study was conducted during the period from March to October 2023. During the outdoor aquaculture period (March–October), the seawater temperatures were 3.6 °C, 8.4 °C, 13.2 °C, 16.8 °C, 22.4 °C, 25.5 °C, 24.3 °C, and 17.6 °C, and the salinity was 30.0, 29.6, 29.6, 29.2, 30.6, 27.6, 29.0, and 30.1, respectively. Nine oysters (two years old) were collected monthly for gill sampling. Total RNA was isolated from samples to determine the mRNA expression levels of *Cg*ALDH6A1.

### 2.4. RNA Interference (RNAi) and Alda-1 Treatment

A total of 54 oysters were randomly divided into six groups: the Blank group (oysters without any treatment), the negative control (NC) group (oysters that received an injection with NC dsRNA), the *Cg*ALDH6A1-RNAi group (oysters injected with *Cg*ALDH6A1 dsRNA), the DMSO group (oysters injected with DMSO), and the Alda-1 group (oysters injected with Alda-1). To interfere with the expression of *Cg*ALDH6A1, specific double-stranded RNA (dsRNA) targeting this gene was designed and synthesized by GenePharma (Shanghai, China). The oysters in the NC group, *Cg*ALDH6A1-RNAi group, DMSO group, and Alda-1 group were injected with the following reagents (100 μL each): 20 μmol negative control (NC) dsRNA, 20 μmol *Cg*ALDH6A1 dsRNA (Table 1), 200 μmol DMSO, and 200 μmol Alda-1 (MCE, Shanghai, China) [47]. Untreated oysters were employed as the Blank group. At 12 h after the injection, the oysters were exposed to 28 °C for 48 h. The gills were collected to analyze the mRNA expression level of *Cg*ALDH6A1 and the measurement of the parameters of oxidative response (the content of MDA and the activities of SOD, CAT, and T-AOC).

### 2.5. RNA Extraction and cDNA Synthesis

Total RNA was extracted from the gills with Trizol reagent (Invitrogen, Carlsbad, CA, USA) per the manufacture’s protocol. First-strand cDNA synthesis was performed using the SevenClever^TM^ First-Strand cDNA Synthesis Kit (with dsDNase) (Seven, Beijing, China). The cDNA mixture was diluted 1:20 (cDNA:ddH_2_O) for use in RT-qPCR analysis.

### 2.6. RT-qPCR Analysis for mRNA Expression

To measure the mRNA expression levels of target *Cg*ALDH genes (*Cg*ALDH1β1, *Cg*ALDH1β2, *Cg*ALDH2α, *Cg*ALDH6A1, *Cg*ALDH7A1, and *Cg*ALDH8A1), RT-qPCR was conducted with corresponding primers (Table 1). RT-qPCR was performed with the SYBR Premix Ex TaqTM Kit (RR420, Takara, Japan) on an ABI 7500 Real-Time Detection System (Thermo Fisher, Waltham, MA, USA). The fragment of elongation factor (*Cg*EF, NM_001305313) was amplified with the primers *Cg*EF-F and *Cg*EF-R and used as internal reference. The 2^−ΔΔCT^ method was applied to calculate the mRNA expression level [48].

### 2.7. Evaluation of Oxidative Stress Response

The enzymatic antioxidants, total antioxidant capacity (T-AOC), and content of MDA in the tissues from the oysters in the blank, NC, *Cg*ALDH6A1-RNAi, DMSO, and Alda-1 groups were analyzed to assess the oxidative stress level following high-temperature stress and the responses to dsRNA (*Cg*ALDH6A1-RNAi) and Alda-1 treatments. The activities of catalase (CAT) and superoxide dismutase (SOD), total antioxidant capacity (T-AOC), malondialdehyde (MDA) content, and total protein content were quantified using the respective assay kits (A007-1-1 for CAT, A001-3-2 for SOD, A015-1-2 for T-AOC, A003-1-2 for MDA, and A045-2-2 for total protein) according to the manufacturer’s protocol (Jiancheng Bioengineering Institute, Nanjing, China).

### 2.8. Histopathological Observation of Tissues

Gill tissues from the blank, NC, *Cg*ALDH6A1-RNAi, DMSO, and Alda-1 groups were obtained for histopathological observation. Each tissue sample was cut into small pieces, about 3 mm square, and then immersed in Bouin’s solution to fix them for 24 h; they were then rinsed with 70% ethanol to remove fixative. Subsequently, the samples underwent sequential dehydration, wax immersion, and paraffin embedding using a paraffin-embedding machine to prepare paraffin sections, which were then dewaxed, stained with hematoxylin–eosin (H&E), re-dehydrated, and mounted with neutral balsam. Finally, they were dehydrated and sealed with neutral gum sealing and then subjected to microscopy, image collation, and analysis.

### 2.9. Data Analysis

Experimental data are presented as means ± standard deviations (n = 3) and were analyzed using one-way ANOVA followed by multiple comparison tests using IBM SPSS 24.0 [49], with * *p* < 0.05, ** *p* < 0.01, and *** *p* < 0.001.

## 3. Results

### 3.1. The ALDH Gene Family in Oysters

A total of 14 ALDH homologues were identified in the genome pertaining to the oysters (Table 2). The ALDH18 subfamily was not detected in a substantially different intensity relative to humans, fruit flies, and razor clams. The homologues’ CDS lengths ranged from 1449 to 2775 bp, encoding 482 to 924 amino acids, with a relative molecular mass ranging from 91.23 to 102.47 kD and an isoelectric point ranging from 5.6 to 8.5.

**Figure 1:**
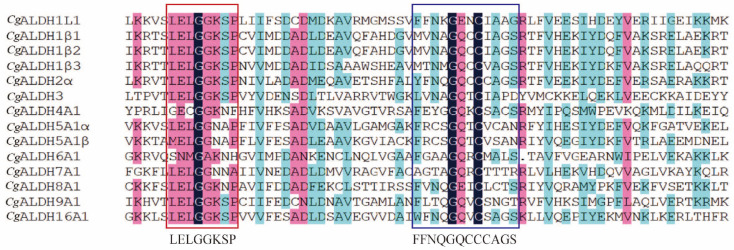
Multiple sequence alignment of *Cg*ALDHs. Black shading indicates identical residues, pink shading indicates similar residues that are more than 80% similar, and blue shading indicates similar residues that are more than 50% similar. The red boxes indicate the conserved glutamic active sites, and the blue boxes indicate the conserved glutamic cysteine active sites.

**Figure 2:**
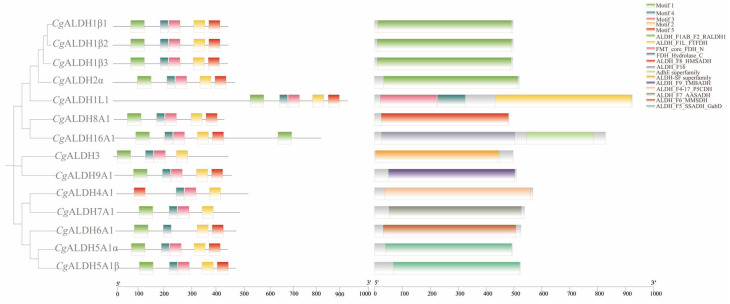
Phylogenetic tree, conserved motifs, and conserved domain of *Cg*ALDH family genes.

**Figure 3:**
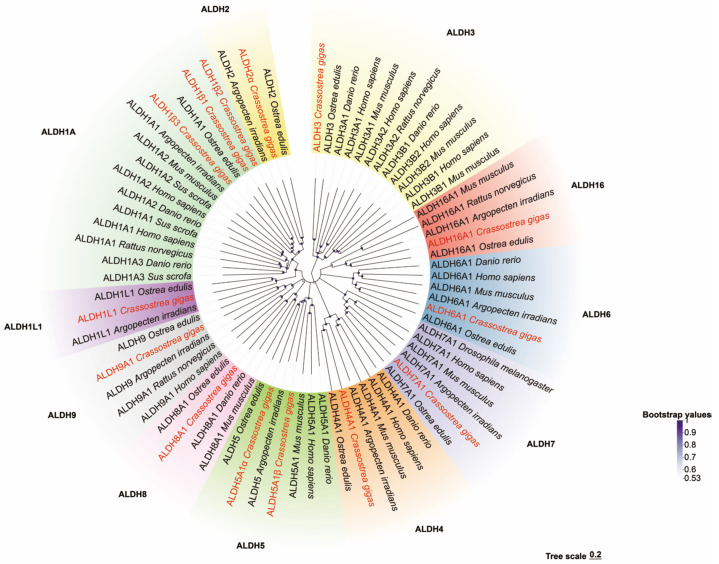
Phylogenetic tree of ALDH family genes.

Multiple-sequence alignment revealed the presence of both glutamate active sites and cysteine active sites in all the *Cg*ALDH genes (Figure 1). Five motifs were identified from these *Cg*ALDHs using MEME online (https://meme-suite.org/meme/ accessed on 21 March 2025), and each ALDH protein contained three to five motifs (Figure 2). Structural domain prediction results revealed that 14 ALDH genes contain at least one complete ALDH domain, and genes from the same subfamily share similar structural characteristics. Among them, the *Cg*ALDH1, *Cg*ALDH2, and *Cg*ALDH5 subfamilies encompassed the ALDH_F1AB_F2_RALDH1 domain, and *Cg*ALDH6A1 possessed the ALDH_F6_MMSDH domain. A phylogenetic tree was constructed using 74 molluscan ALDH proteins, and the ALDHs from the same subfamily were clustered together (Figure 3).

The phylogenetic tree shows that these genes have aggregated into two distinct evolutionary branches. *Cg*ALDH3 was found to be associated with a single cluster, while *Cg*ALDH1, *Cg*ALDH2, *Cg*ALDH4, *Cg*ALDH5, *Cg*ALDH6, *Cg*ALDH7, *Cg*ALDH8, *Cg*ALDH9, and *Cg*ALDH16 formed a distinct evolutionary branch. Within this branch, the families of both *Cg*ALDH1 and *Cg*ALDH2, as well as *Cg*ALDH8 and *Cg*ALDH9, showed a close phylogenetic relationship, while the *Cg*ALDH3 subfamily formed a cluster distant from the other gene subfamilies. All *Cg*ALDH genes clustered closely with similar genes from other known species, with a primary alignment with the homologous genes from the European flat oyster, *Ostrea edulis*.

### 3.2. Transcriptome Heatmap Analysis and the Expression Profiles of CgALDHs in Tissues and Under High-Temperature Stress

**Figure 4:**
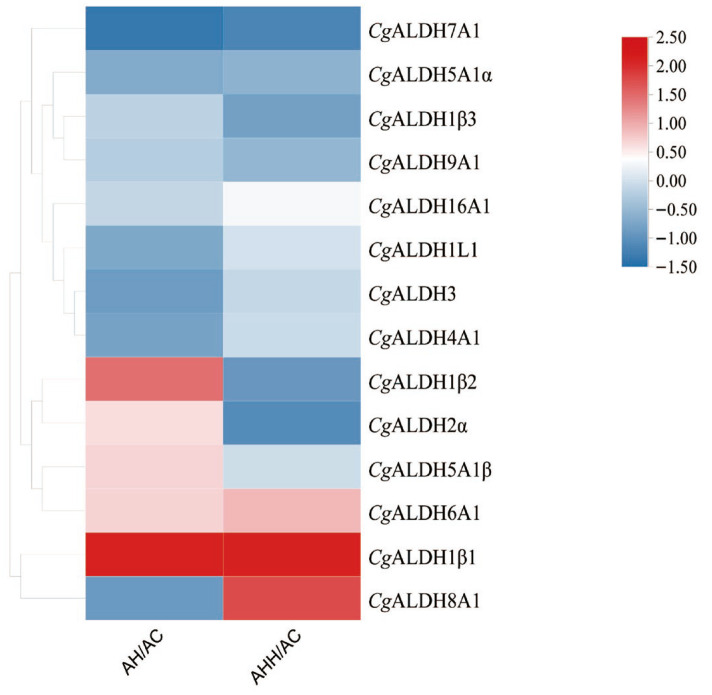
Heat map of mRNA expression of *Cg*ALDHs under high-temperature stress. Each cell in the heat map corresponds to the ratio of FPKM between the high-temperature stress sample and blank sample. The intensity of the color from red to blue indicates the magnitude of differential expression. The AH group refers to oysters subjected to 28 °C high-temperature stress for 12 h; the AHH group refers to those exposed to 35 °C high-temperature stress for 12 h; and the AC group is the untreated control.

Heat map analysis was conducted on the high-temperature stress groups and control group using the transcriptomic FPKM data (Figure 4). Following exposure to high-temperature stress at 28 °C, the expression levels of *Cg*ALDH1β1, *Cg*ALDH1β2, *Cg*ALDH2α, and *Cg*ALDH6A1 in the gills were 0.62-fold, 0.46-fold, 0.25-fold, and 0.27-fold higher than those in the AC group, respectively (*p* < 0.05). Conversely, the expression levels of *Cg*ALDH7A1 and *Cg*ALDH8A1 in the gills were significantly down-regulated by 24% and 12% compared with those in the AC group, respectively (*p* < 0.05). Additionally, 12 h after high-temperature stress at 35 °C, the expression levels of *Cg*ALDH1β1, *Cg*ALDH6A1, and *Cg*ALDH8A1 in the gills were 0.52-fold, 0.21-fold, and 0.43-fold higher than those in the AC group, respectively (*p* < 0.05). Meanwhile, the expression levels of *Cg*ALDH1β2, *Cg*ALDH2α, and *Cg*ALDH7A1 in the gills were significantly down-regulated by 25%, 29%, and 31% compared with those in the AC group, respectively (*p* < 0.05).

**Figure 5:**
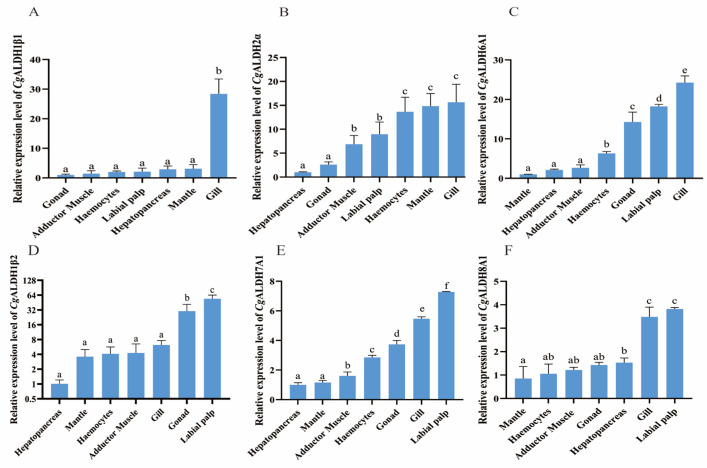
The mRNA expression patterns of *Cg*ALDHs in different tissues under 28 °C high-temperature stress for 24 h. (**A**): *Cg*ALDH1β1; (**B**): *Cg*ALDH2α; (**C**): *Cg*ALDH6A1; (**D**): *Cg*ALDH1β2; (**E**): *Cg*ALDH7A1; (**F**): *Cg*ALDH8A1. Vertical bars represent the mean ± S.D. (n = 3). Significant differences between groups are indicated by different letters (*p* < 0.05).

The mRNA expression levels of six *Cg*ALDHs, including *Cg*ALDH1β1, *Cg*ALDH1β2, *Cg*ALDH2α, *Cg*ALDH6A1, *Cg*ALDH7A1, and *Cg*ALDH8A1, in several tissues were analyzed using RT-qPCR. The six *Cg*ALDH genes were found to be ubiquitously expressed in all the examined tissues with different expression profiles. *Cg*ALDH1β1 showed the highest expression levels in the gills and the lowest in the gonads (Figure 5A). The expression level of *Cg*ALDH1β1 in the gills was 26.74-fold higher than that in the gonad (*p* < 0.001), with no significant differences observed in the mantle, hepatopancreas, labial palp, or hemocytes. *Cg*ALDH1β2 exhibited the highest expression level in the labial palp and the lowest in the hepatopancreas (Figure 5B). The expression levels of *Cg*ALDH1β2 in the labial palp and gonad were 52.56-fold (*p* < 0.001) and 29.00-fold (*p* < 0.001) higher than that in the hepatopancreas, respectively, with no significant difference in the gills, adductor muscle, hemocytes, or mantle. *Cg*ALDH2α showed the highest expression level in the gills and the lowest in the hepatopancreas (Figure 5C). The expression level of *Cg*ALDH2α in the gills, mantle, hemocytes, labial palp, and adductor muscle was 14.59-fold (*p* < 0.001), 13.78-fold (*p* < 0.001), 12.59-fold (*p* < 0.001), 7.93-fold (*p* < 0.05), and 5.86-fold (*p* < 0.05) higher than that in the hepatopancreas, respectively, with no significant difference in the gonad. *Cg*ALDH6A1 displayed the highest expression level in the gills and the lowest in the mantle (Figure 5D). The expression level of *Cg*ALDH6A1 in the gills, labial palp, gonad, and hemocytes was 23.24-fold (*p* < 0.001), 17.22-fold (*p* < 0.001), 13.29-fold (*p* < 0.001), and 5.30-fold (*p* < 0.001) higher than that in the mantle, respectively, with no significant difference in the hepatopancreas or adductor muscle. *Cg*ALDH7A1 showed the highest expression level in the labial palp and the lowest in the hepatopancreas (Figure 5E). The expression level of *Cg*ALDH7A1 in the labial palp, gills, gonads, hemocytes, and adductor muscle was 6.24-fold (*p* < 0.001), 4.44-fold (*p* < 0.001), 2.71-fold (*p* < 0.001), 1.84-fold (*p* < 0.001), and 0.90-fold (*p* < 0.05) higher than that in the hepatopancreas, respectively, with no significant differences in the mantle. *Cg*ALDH8A1 exhibited the highest expression level in the labial palp and the lowest in the mantle (Figure 5F). The expression levels of *Cg*ALDH8A1 in the labial palp, gills, hepatopancreas, gonads, adductor muscle, and hemocytes were 6.62-fold (*p* < 0.001), 5.95-fold (*p* < 0.001), 2.05-fold (*p* < 0.001), 1.86-fold (*p* < 0.001), 1.43-fold (*p* < 0.05), and 1.11-fold (*p* < 0.05) higher than those in the mantle, respectively.

**Figure 6:**
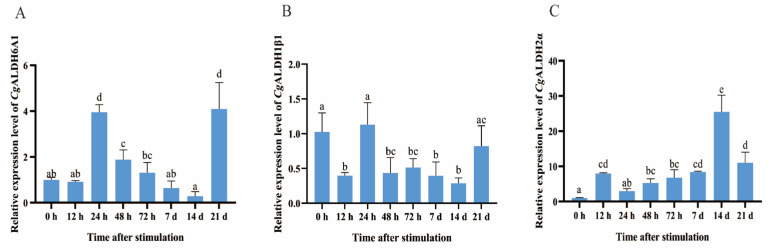
The mRNA expression levels of six *Cg*ALDHs in gills after exposure to 28 °C. (**A**): *Cg*ALDH6A1; (**B**): *Cg*ALDH1β1; (**C**): *Cg*ALDH2α. Vertical bars represent the mean ± S.D. (n = 3). Significant differences between groups are indicated by different letters (*p* < 0.05).

The expression levels of *Cg*ALDH1β1, *Cg*ALDH2α, and *Cg*ALDH6A1 in the gills of oysters at different time points after undergoing high-temperature stress at 28 °C were further examined via RT-qPCR. After the oysters were subjected to high-temperature stress at 28 °C, the expression level of *Cg*ALDH1β1 decreased significantly, by 50–72%, at 12 h, 48 h, 72 h, 7 d, and 14 d, with respective reductions of 61% (*p* < 0.01), 57% (*p* < 0.01), 50% (*p* < 0.05), 61% (*p* < 0.01), and 72% (*p* < 0.001) compared with that at 0 h. In contrast, the expression of *Cg*ALDH2α increased significantly at 12 h, 48 h, 72 h, 7 d, 14 d, and 21 d, being 6.97-fold (*p* < 0.01), 4.27-fold (*p* < 0.05), 5.76-fold (*p* < 0.01), 7.38-fold (*p* < 0.001), 23.40-fold (*p* < 0.001), and 10.05-fold (*p* < 0.001) higher than that at 0 h, respectively. The expression of *Cg*ALDH6A1 exhibited a pattern of first increasing and then decreasing. At 24 h and 21 d after exposure to high-temperature stress at 28 °C, its expression increased significantly, being 2.94-fold (*p* < 0.001) and 3.08-fold (*p* < 0.001) higher than that at 0 h, respectively (Figure 6).

### 3.3. The mRNA Expression Level of CgALDH6A1 During Outdoor Aquaculture

**Figure 7:**
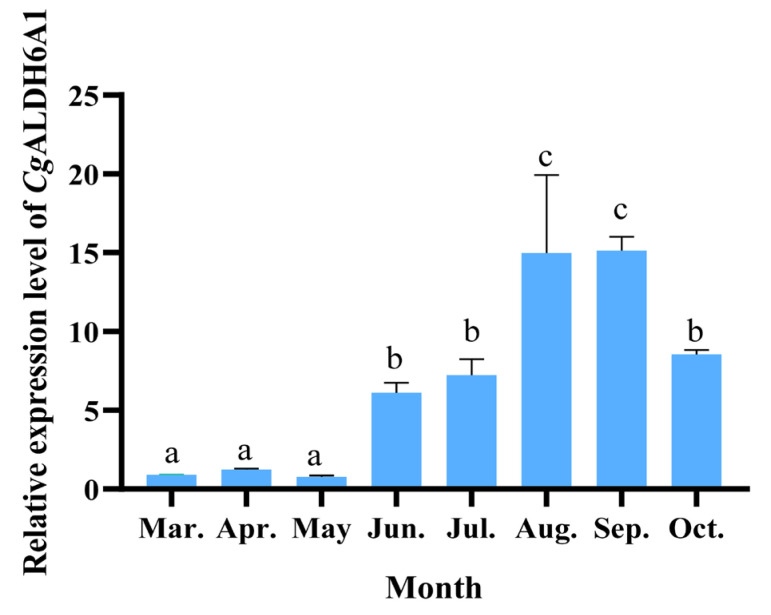
The mRNA expression levels of *Cg*ALDH6A1 in gill tissue of oysters during the aquaculture period. Vertical bars represent the mean ± S.D. (n = 3). Significant differences between groups are indicated by different letters (*p* < 0.05).

As the expression of *Cg*ALDH6A1 in the gills increased significantly after exposure to high-temperature stress, its alteration in the gills of oysters during an outdoor aquaculture period was examined to further investigate the involvement of *Cg*ALDH6A1 in the response to high-temperature stress in aquaculture. The expression level of *Cg*ALDH6A1 in June, July, August, September, and October was significantly higher than that in March, being 5.78-fold (*p* < 0.01), 7.03-fold (*p* < 0.001), 15.67-fold (*p* < 0.001), 15.82-fold (*p* < 0.001), and 8.50-fold (*p* < 0.001) greater, respectively (Figure 7).

### 3.4. The Changes in Oxidative Stress Response and Histopathological Changes in CgALDH6A1-RNAi in Oysters

**Figure 8:**
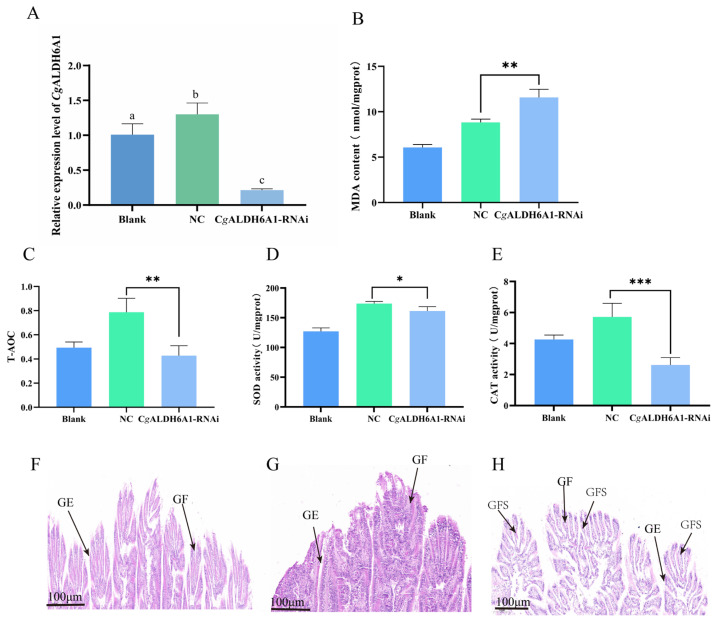
The mRNA expression level of *Cg*ALDH6A1 and changes in oxidative stress indexes in gills of *Cg*ALDH6A1-RNAi oysters after being subjected to high-temperature stress. The blank group consisted of untreated oysters; the NC group included oysters that were injected with negative control (NC) dsRNA; and the oysters in *Cg*ALDH6A1-RNAi group were injected with *Cg*ALDH6A1 dsRNA. (**A**): *Cg*ALDH6A1 mRNA expression level. (**B**): MDA content. (**C**): T-AOC. (**D**): SOD activity. (**E**): CAT activity. (**F**): Blank group, (**G**): NC group, (**H**): *Cg*ALDH6A1 RNAi group; GE: gill epithelium, GF: gill filament, GFS: gill filament swelling. Significant differences between groups are indicated by different letters (*p* < 0.05). The significant difference between the control group and the experimental group is represented by asterisks (*: *p* < 0.05, **: *p* < 0.01, ***: *p* < 0.001).

After the expression of *Cg*ALDH6A1 was suppressed by RNAi, the changes in oxidative stress response including with respect to MDA content, T-AOC, and the activities of CAT and SOD in the gills were examined to further confirm the involvement of *Cg*ALDH6A1 in the high-temperature stress response. The mRNA expression of *Cg*ALDH6A1 in the gills decreased significantly 48 h after the injection of targeted dsRNA, with a fold change equal to 0.16 (*p* < 0.001) of that in the NC group (Figure 8), indicating that the expression of *Cg*ALDH6A1 was suppressed effectively. The MDA content in *Cg*ALDH6A1-RNAi oyster gills significantly increased (1.31-fold, *p* < 0.01) relative to that in the NC group, while T-AOC and the activities of SOD and CAT in the gills all decreased significantly, with 0.54-fold (*p* < 0.01), 0.93-fold (*p* < 0.05), and 0.45-fold (*p* < 0.001) reductions relative to the NC group, respectively (Figure 8B–E). Gill tissues from the oysters in the *Cg*ALDH6A1-RNAi group were collected and observed under a microscope after being stained with hematoxylin–eosin. The gill epithelium of the blank group exhibited an orderly arrangement in a single layer, with clearly visible nuclei that were round or oval, while the gill filaments of *Cg*ALDH6A1-RNAi individuals were disorganized, with obvious swelling (Figure 8F–H).

### 3.5. Changes in the Oxidative Stress Response After the Injection of Alda-1 In Vivo

**Figure 9:**
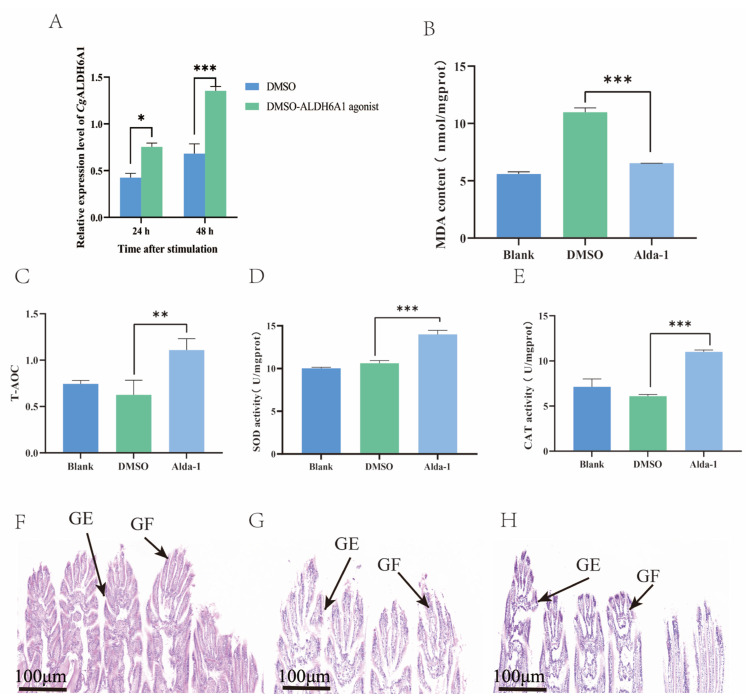
The mRNA expression level of *Cg*ALDH6A1 and changes in oxidative stress indexes in gills after the injection of agonist in vivo after exposure to high-temperature stress. (**A**): *Cg*ALDH6A1 mRNA expression level. (**B**): MDA content. (**C**): T-AOC. (**D**): SOD activity. (**E**): CAT activity. (**F**): Blank group, (**G**): DMSO group, (**H**): Alda-1 group; GE: gill epithelium, GF: gill filament. Vertical bars represent the mean ± S.D. (n = 3). Asterisks indicate significant differences (*: *p* < 0.05; **: *p* < 0.01; ***: *p* < 0.001).

After *Cg*ALDH6A1 expression was activated with Alda-1 injection, the changes in the oxidative stress response, including MDA content, T-AOC, and the activities of CAT and SOD, in the gills were examined to further confirm the involvement of *Cg*ALDH6A1 in the high-temperature stress response. The mRNA expression of *Cg*ALDH6A1 in the gills increased significantly 24 h and 48 h after the injection of Alda-1, being 1.78-fold (*p* < 0.05) and 1.98-fold (*p* < 0.001) of that in the DMSO group, respectively (Figure 9A), indicating that the expression *Cg*ALDH6A1 was activated effectively. The most significant change in the expression level of *Cg*ALDH6A1 was observed 48 h after agonist injection. Thus, the changes in oxidative stress indexes were explored. The Alda-1 group had a significantly reduced gill MDA content (0.59-fold of the DMSO group, *p* < 0.001), whereas its gill T-AOC (1.79-fold, *p* < 0.01), SOD activity (1.33-fold, *p* < 0.001), and CAT activity (1.81-fold, *p* < 0.001) were all significantly higher than those in the DMSO group (Figure 9B–E). Gill tissues of oysters in the Alda-1 group were collected, stained with hematoxylin–eosin (HE), and then observed under a microscope. The gill epithelium of the blank group exhibited an orderly arrangement in a single layer, with clearly visible nuclei that are round or oval, and the gills from the Alda-1 group showed no obvious morphological changes in comparison with the blank group (Figure 9F–H).

## 4. Discussion

Intertidal organisms, including oysters, have evolved highly conserved molecular regulatory strategies to cope with environmental stress [50]. ALDH plays an important role in the process by cooperating with the antioxidant system in alleviating oxidative stress under high-temperature stress. In this study, we found that *Cg*ALDH6A1-targeted MDA mediates the oxidative stress response of oysters, with the aim of elucidating its role in regulating antioxidant function under high-temperature stress.

A total of 14 ALDH family members were identified in the oyster genome, exceeding the 11 ALDH genes reported in fruit flies [19,20] but amounting to less than the 19 and 16 ALDHs identified in humans [51] and razor clams [21], respectively. This pattern suggests that the *Cg*ALDH family has undergone moderate gene expansion during invertebrate evolution. Results from structural-domain prediction indicated that ALDH genes within the same subfamily share similar structural characteristics. Among them, *Cg*ALDH6A1 contains the ALDH_F6_MMSDH domain and oxidizes various aliphatic and aromatic aldehydes [52]. ALDH6A1, also known as methylmalonate semialdehyde (MMS) dehydrogenase, is the only known human ALDH isoform that depends on coenzyme A (CoA) for its catalytic activity, as it metabolizes methylmalonate semialdehyde and converts lipid-peroxidation-derived aldehydes, such as MDA, into acetyl-CoA. The degradation of MDA by other ALDH isoforms proceeds through NAD(P)^+^-dependent oxidative reactions, ultimately leading to the decomposition of MDA into carbon dioxide and water. Phylogenetic analysis showed that the ALDH1 and ALDH2 subfamilies, as well as *Cg*ALDH8 and *Cg*ALDH9, cluster into the same phylogenetic branch, suggesting a close evolutionary relationship. The *Cg*ALDH genes primarily cluster with homologs from the European flat oyster, forming a sister clade with the bay scallop *Argopecten irradians*, indicating evolutionary conservation and potential functional significance of ALDH genes across bivalve lineages. Conservative site analysis showed that *Cg*ALDH proteins possess both a glutamate-active site (LELGGKSP) and a cysteine-active site (FFNQGQCCCAGS), which are essential for ALDH catalytic function [53]. These conserved sequences highlight the evolutionary stability and structural importance of ALDH catalytic domains across species.

As key aldehyde-detoxifying enzymes, ALDHs maintain cellular homeostasis and play essential roles in stress responses [54,55,56]. In this study, six *Cg*ALDH genes (*Cg*ALDH1β1, *Cg*ALDH1β2, *Cg*ALDH2α, *Cg*ALDH6A1, *Cg*ALDH7A1, and *Cg*ALDH8A1) were found to be involved in high-temperature stress based on transcriptomic data obtained under high-temperature stress. The expression of some *Cg*ALDHs was almost specific to a certain tissue (e.g., *Cg*ALDH1β1 in the gills), while others were expressed in all tissues, with only slight differences among tissues. Notably, the hepatopancreas, a detoxification tissue, showed high expression only for *Cg*ALDH8A1, warranting further investigation. It was found that *Cg*ALDH1β1, *Cg*ALDH2α, and *Cg*ALDH6A1 mRNA levels were highly expressed in the gills. Gill tissue is the main tissue responsible for triggering a response to high-temperature stress, and it achieves this response by synthesizing heat shock proteins, amplifying antioxidant capacity, and governing apoptosis [57]. Thus, the expression levels of the three *Cg*ALDHs after high-temperature stress were examined. In response to high-temperature stress, the expression of *Cg*ALDH6A1 and *Cg*ALDH2α in the gills significantly upregulated, and *Cg*ALDH6A1 was chosen for the further investigation. Monthly expression profiles of *Cg*ALDH6A1 during the outdoor aquaculture period revealed a significant increase around summer from June to October. Oysters exhibit antioxidant responses to thermal stress, and previous studies have reported increased ALDH activity under such conditions [31]. Similarly, high temperatures induce oxidative stress in shrimp, triggering increased ALDH expression to detoxify accumulated aldehydes. These results indicate that ALDH6A1 is responsive to high-temperature stress and plays a role in regulating antioxidant activity in response to stress.

High-temperature stress induces oxidative stress damage in oysters through elevated MDA accumulation [58]. Traditionally, this is mitigated by antioxidant enzymes such as SOD and CAT, which reduce MDA levels under stress [59]. ALDH enzymes also contribute to this defense by converting toxic aldehydes into non-toxic acids, thereby alleviating oxidative stress [54]. For instance, ALDH1A1 mediates the conversion of 3,4-dihydroxyphenylacetaldehyde into 3,4-dihydroxyphenylacetic acid in neuronal tissues [60], whereas ALDH2 is essential for detoxifying aldehydes derived from lipid peroxidation, including 4-HNE and MDA [29]. In this study, MDA content in the gills of *Cg*ALDH6A1-RNAi group oysters significantly increased under 28 °C exposure, while SOD activity, CAT activity, and T-AOC were all significantly decreased. Conversely, MDA content in Alda-1 group significantly decreased, while SOD activity, CAT activity, and T-AOC all significantly increased, suggesting that *Cg*ALDH6A1 functions as an antioxidant enzyme by degrading MDA. This is consistent with previous observations that ALDH7B4 degrades MDA under thermal stress [29] and *Os*ALDH7 participates in MDA detoxification during oxidative stress in desiccated rice seeds [61]. Histological analysis showed that *Cg*ALDH6A1-RNAi oysters exhibited pronounced gill damage, including filament swelling, while Alda-1-injected oysters showed slight damage, suggesting a functional link between *Cg*ALDH6A1 and the antioxidant system. Similar results have been reported for mice, where elevated ALDH activity restores oxidative stress levels through the activation of SOD and CAT and the lowering of MDA [62] and the activation of ALDH2 results in a significant increase in the activities of CAT and SOD and a significant decrease in MDA content [63]. Previous studies have reported that antioxidant mechanisms trigger prior to the formation of MDA, mainly by neutralizing reactive oxygen species (ROS) through specific chemical reactions to alleviate oxidative damage in organisms and indirectly reduce MDA content [64]. As aldehyde dehydrogenases, ALDHs are able to decompose aldehydes such as MDA to mitigate oxidative damage. These results collectively indicate that *Cg*ALDH6A1 cooperates with the antioxidant system to jointly alleviate oxidative stress under high-temperature stress.

High temperatures frequently induce oxidative stress and even summer mortality in oysters. Current prevention strategies, including health assessment, environmental monitoring, and screening of disease-resistant strains, remain limited because shellfish health evaluation still depends largely on morphological observation and physiological–biochemical assays [65]. Several indicators related to oxidative damage and antioxidant capacity (e.g., LPS, LTA, MDA, SOD, CAT, and HSPs) have been proposed for early disease warning for aquaculture animals [66,67]. Our study demonstrates that *Cg*ALDH6A1 mitigates high-temperature stress in oysters by degrading MDA and interacting with the antioxidant system. Its expression level correlates strongly with oxidative damage and antioxidant capacity, suggesting its potential as an early molecular marker for oxidative stress. This study focused on a single species (the Pacific oyster) under controlled laboratory conditions, constraining the generalizability of the findings to other species or field settings. Future research should expand validation under realistic environmental conditions and further assess the applicability of *Cg*ALDH6A1 as a molecular marker. These efforts will support the development of an integrated, multi-target early-warning and management system for sustainable oyster aquaculture.

## 5. Conclusions

In conclusion, 14 ALDH homologues were identified in the genome of the Pacific oyster, among which *Cg*ALDH6A1 responded to high-temperature stress. The MDA content in the gills increased significantly after *Cg*ALDH6A1 was inhibited via dsRNA injection, while T-AOC and the activities of CAT and SOD all decreased significantly. Meanwhile, the gill filaments were observed to be disorganized with obvious swelling under a microscope. The activation of *Cg*ALDH6A1 led to a decrease in gill MDA content, accompanied by significant increases in T-AOC, SOD activity, and CAT activity. However, no obvious morphological change was observed in the gills. These results demonstrate that *Cg*ALDH6A1 mediated oxidative stress responses under high-temperature stress through MDA degradation.

## Figures and Tables

**Table 1:** Information on experimental primers.

Gene Accession Number	Primer Name	Sequence (5′-3′)
Primers for RT-PCR		
XM_011452173.3	*Cg*ALDH2α-F	GGGGAGATTGGGGAATTGACA
	*Cg*ALDH2α-R	TAATTGGCTGGGGAAGCGAT
XM_011428286.4	*Cg*ALDH7A1-F	GCAGCATTAGAGGAGGCCAA
	*Cg*ALDH7A1-R	TTCAGGACAATGGGGGCATC
XM_011441203.3	*Cg*ALDH6A1-F	GCTGGTCAAAGAGGCTGGAT
	*Cg*ALDH6A1-R	TCTTGGCTCCCATGTTGGAC
XM_011438515.4	*Cg*ALDH1β2-F	GCTTCCATCAACATTAAGCGCA
	*Cg*ALDH1β2-R	GCCTCGTCCAGATCAGCATC
XM_011456447.4	*Cg*ALDH8A1-F	GCACAGCAGATGGTTCATTGAT
	*Cg*ALDH8A1-R	CACAACAAGCTAACGCAGCAT
XM_066088776.1	*Cg*ALDH1β1-F	TGACGTTCACGCAGGGTGT
	*Cg*ALDH1β1-R	CTAGGCCGGACATCTTGAACC
NM_001305313	*Cg*EF-F	AGTCACCAAGGCTGCACAGAAAG
	*Cg*EF-R	TCCGACGTATTTCTTTGCGATGT
Primers for gene cloning		
XM_011441203.3	*Cg*ALDH6A1-F1	AACCCCCGAAAACCTTGACA
	*Cg*ALDH6A1-R1	TGGAAACATCTGCTGCCCTT
Primers for RNA interference		
XM_011441203.3	*Cg*ALDH6A1-SI-F	GGUGGAUGCUGAAGGAGAUTT
	*Cg*ALDH6A1-SI-R	AUCUCCUUCAGCAUCCACCTT
	NC-F	UUCUCCGAACGUGUCACGUTT
	NC-R	ACGUGACACGUUCGGAGAATT

**Table 2:** The information on the *Cg*ALDH gene family.

Gene Name	Gene Symbol	mRNA (bp)	Protein (aa)	CDS (bp)	PI	MW (kDa)
*Cg*ALDH1β1	LOC105334905	2253	497	1494	5.92	53.67
*Cg*ALDH1β2	LOC105334906	2009	495	1488	6.07	53.55
*Cg*ALDH1β3	LOC105332228	2449	495	1488	6.08	27.44
*Cg*ALDH1L1	LOC105347685	4173	924	2775	5.79	102.47
*Cg*ALDH2α	LOC105344389	2346	519	1560	6.18	53.65
*Cg*ALDH3	LOC105330234	2793	497	1494	8.47	55.73
*Cg*ALDH4A1	LOC105347830	1976	568	1707	8.48	63.69
*Cg*ALDH5A1α	LOC105346453	2569	494	1485	6.03	52.51
*Cg*ALDH5A1β	LOC105317861	2357	523	1572	6.25	57.13
*Cg*ALDH6A1	LOC105336766	1999	525	1578	6.48	57.24
*Cg*ALDH7A1	LOC105327683	1750	538	1617	7.04	58.26
*Cg*ALDH8A1	LOC105347377	4475	482	1449	6.25	53.32
*Cg*ALDH9A1	LOC105326193	2184	509	1530	5.61	55.60
*Cg*ALDH16A1	LOC105334225	6012	828	2287	6.45	91.23

## Data Availability

The original contributions presented in this study are included in the article/Supplementary Material. Further inquiries can be directed to the corresponding authors.

## Supplementary Materials

The following supporting information can be downloaded at https://www.mdpi.com/article/10.3390/antiox14121423/s1, Table S1: The ALDH sequences of model organisms used in this study.

## Abbreviations

The following abbreviations are used in this manuscript:

ALDH	aldehyde dehydrogenase
MDA	malondialdehyde
SOD	superoxide dismutase
CAT	catalase
T-AOC	total antioxidant capacity
4-HNE	4-hydroxynonenal
