# Supplementary material for: The Regulatory Role of CgALDH6A1 in the Oxidative Stress Response of Crassostrea gigas Under High-Temperature Stress
Source: Antioxidants (Basel). 2025 Nov 27;14(12):1423. doi: 10.3390/antiox14121423 (PMC12729558; doi:10.3390/antiox14121423)
Supplement: Supplementary file 1 [file antioxidants-14-01423-s001.zip › antioxidants-3964259-supplementary.pdf]

Table S1 The ALDH sequences of model organisms used in this study.

Species	GenBank ID	Species	GenBank ID
ALDH1A1		*Danio rerio*	NP_775328.2
Rattus norvegicus	XP_038949859.1	ALDH3B2	
Homo sapiens	NP_000680.2	*Homo sapiens*	NP_001026786.3
Sus scrofa	XP_020920570.1	*Mus musculus*	NP_001170909.1
ALDH1A2		ALDH4A1	
Homo sapiens	NP_001193826.1	*Homo sapiens*	NP_001154976.1
Sus scrofa	XP_020950265.1	*Mus musculus*	NP_780647.3
Danio rerio	NP_571925.1	Danio rerio	NP_957452.1
Mus musculus	NP_033048.2	ALDH5A1	
ALDH1A3		*Homo sapiens*	NP_001071.1
Sus scrofa	XP_020954233.1	*Mus musculus*	NP_766120.1
Danio rerio	NP_001038210.1	Danio rerio	NP_001103938.2
ALDH1B1		ALDH6A1	
Homo sapiens	NP_000683.3	*Homo sapiens*	NP_001265522.1
Mus musculus	NP_082546.1	*Mus musculus*	NP_001300896.1
Sus scrofa	XP_003353634.1	Danio rerio	NP_001002374.1
ALDH1L1		ALDH7A1	
Danio rerio	NP_001185701.1	*Homo sapiens*	NP_001173.2
ALDH1L2		*Mus musculus*	NP_001120810.1
Danio rerio	XP_002661418.2	*Drosophila melanogaster*	NP_649099.1
ALDH2		ALDH8A1	
Homo sapiens	NP_000681.2	*Mus musculus*	NP_848828.1
Mus musculus	NP_001295379.1	Danio rerio	NP_001004540.1
Sus scrofa	NP_001038076.1	ALDH9A1	
ALDH3A1		*Homo sapiens*	NP_000687.3
Homo sapiens	NP_000682.3	*Rattus norvegicus*	NP_071609.2
Mus musculus	NP_001106196.1	ALDH16A1	
Danio rerio	XP_001335979.2	*Mus musculus*	NP_666066.1
ALDH3A2		*Rattus norvegicus*	XP_006229162.1
Homo sapiens	NP_000373.1	ALDH18A1	
Rattus norvegicus	NP_113919.2	*Homo sapiens*	NP_001017423.1
ALDH3B1		*Mus musculus*	NP_062672.2
Homo sapiens	NP_000685.1	*Rattus norvegicus*	NP_001101994.1
Mus musculus	NP_080592.2		
